# Assessment of Day-7 Postexposure Testing of Asymptomatic Contacts of COVID-19 Patients to Evaluate Early Release from Quarantine — Vermont, May–November 2020

**DOI:** 10.15585/mmwr.mm7001a3

**Published:** 2021-01-08

**Authors:** Amanda Jones, Veronica Fialkowski, Lauren Prinzing, Jeffrey Trites, Patsy Kelso, Mark Levine

**Affiliations:** 1 Vermont Department of Health.

On May 8, 2020, the Vermont Department of Health (VDH) issued a Health Update* recommending shortening the duration of quarantine for persons exposed to SARS-CoV-2, the virus that causes coronavirus disease 2019 (COVID-19). Exposed persons who were in quarantine could be tested by polymerase chain reaction (PCR) on or after quarantine day 7. Those who had remained asymptomatic throughout quarantine and who received a negative SARS-CoV-2 PCR test result on or after day 7 could end quarantine. This policy was based on a report suggesting that symptom onset occurs within this time frame in approximately three quarters of COVID-19 cases (*1*) and on consultation of the Vermont Health Commissioner with the U.S. Surgeon General. VDH implemented this policy to minimize restrictions on state residents, recognizing that some reduction could occur in the prevention benefit of quarantine to contain the spread of SARS-CoV-2. State-run SARS-CoV-2 testing sites were made available to increase access to no-cost testing and facilitate implementation of this policy. During August 1–December 1, among persons seeking testing at a VDH SARS-CoV-2 testing site, 36% stated that their reason for seeking testing was to end quarantine early (VDH, unpublished data, December 7, 2020), indicating that persons were aware of and following the policy and using the testing services provided. To assess the effectiveness of this policy, VDH analyzed testing data for contacts of persons with a COVID-19 diagnosis. During May 8–November 16, VDH identified 8,798 exposed contacts of COVID-19 patients; 3,983 (45%) had sought testing within 14 days of their exposure, with day 0 defined as the date of last exposure noted in the case investigation record. Among these persons, 2,200 (55%) who received testing on days 7–10 were included in this analysis; 977 (44.9%) of these contacts had a specimen collected for testing on day 7. Among these, 34 (3%) had test results that were positive, 940 (96%) had results that were negative, and three (<1%) had results that were indeterminate (Table). Among the 34 contacts who received a positive SARS-CoV-2 PCR test result on day 7 after exposure, 12 (35%) were asymptomatic. The remaining 22 contacts with positive test results were symptomatic at the time of testing; approximately one half had developed symptoms on days 4–7 after exposure. Among the 940 contacts who received negative test results on specimens collected on day 7 after exposure, 154 (16%) had a subsequent test within the next 7 days (i.e., days 8–14); among these, 152 (99%) had tests that remained negative, and two (1%) had results that were indeterminate.

**TABLE:** Polymerase chain reaction (PCR) testing results among persons tested for SARS-CoV-2 during quarantine,* by quarantine day — Vermont, May 8, 2020–November 16, 2020

PCR test result	No. (%) of test results	
	Quarantine day 7	Quarantine day 8–10
**All contacts tested days 7–10**	977 (100)	1,223 (100)
Positive	34 (3)	53 (4)
Negative	940 (96)	1,159 (95)
Indeterminate	3 (<1)	11 (1)
**Contacts who retested after negative or indeterminate results** ^†^	154 (15.7)	108 (8.8)
Positive^§^	0 (—)	0 (—)
Negative^§^	152 (99)	107 (99)
Indeterminate^§^	2 (1)	1 (1)

* The quarantine period begins with the date of last exposure to a person with a positive test result for SARS-CoV-2.

^†^ Percentage of all persons tested.

^§^ Percentage of persons retested.

In addition to the 977 persons who received testing on day 7 after exposure, 1,223 (55.1% of all contacts tested on days 7–10 postexposure) had a specimen collected for SARS-CoV-2 PCR testing on day 8, 9, or 10. Among those persons, 53 (4%) had test results that were positive, 1,159 (95%) had results that were negative, and 11 (1%) had results that were indeterminate. Among the 53 contacts who received a positive SARS-CoV-2 PCR result on specimens collected days 8–10 after exposure, 12 (23%) were asymptomatic (mean time since symptom onset = 6 days). Among the 1,170 contacts who received a negative or indeterminate test result on specimens collected on days 8–10 after exposure, 108 (9%) had a subsequent specimen tested; 107 (99%) remained negative, and one (1%) had a result that was indeterminate. Therefore, among all 2,200 contacts tested on days 7–10 after exposure, 87 (4%) persons had a positive test result, 24 (28%) of whom were asymptomatic.

The findings in this report are subject to at least three limitations. First, this analysis was conducted on a convenience sample that included only positive test results received electronically or by fax and negative test results received electronically. Negative test results that were received by fax were not entered into the surveillance system and so are not included in this analysis. Second, the limited demographic data on contacts of patients could hamper the matching of a contact with a laboratory result; thus, the number of contacts tested might be underreported. Although persons who were retested remained negative (1% indeterminate), they represented only 16% of the total number of persons who discontinued quarantine. Finally, asymptomatic status during quarantine until the time of testing for persons testing negative could not be verified.

These results indicate that among the persons in quarantine who tested negative at day 7 after exposure, none who were retested between day 8 and 14 were positive. Allowing asymptomatic persons to shorten quarantine with a negative test at day 7 or later has not been demonstrated to result in transmission of SARS-CoV-2, indicating that the policy has been effective. These results also indicate that 3.9% of contacts tested on days 7–10 after exposure were infected with SARS-CoV-2. In addition to reducing the duration of quarantine for exposed contacts, Vermont’s policy might have provided additional benefits to the state’s pandemic response by identifying some asymptomatic patients earlier in the course of their illness through enhancing statewide surveillance testing of an exposed group. This assessment supports Vermont’s policy as being effective and offers data to support recommendations to shorten quarantine with testing such as those provided in CDC’s updated quarantine guidance.^†^

## Footnotes

* https://www.healthvermont.gov/sites/default/files/documents/pdf/HAN-COVID-19-ChangetoQuarantine.pdf.

† https://wwwdev.cdc.gov/coronavirus/2019-ncov/more/scientific-brief-options-to-reduce-quarantine.html.
